# The Costs and Benefits of Two Secondary Symbionts in a Whitefly Host Shape Their Differential Prevalence in the Field

**DOI:** 10.3389/fmicb.2021.739521

**Published:** 2021-09-30

**Authors:** Hong-Wei Shan, Shu-Sheng Liu

**Affiliations:** ^1^State Key Laboratory for Managing Biotic and Chemical Threats to the Quality and Safety of Agro-products, Key Laboratory of Biotechnology in Plant Protection of Ministry of Agriculture and Zhejiang Province, Institute of Plant Virology, Ningbo University, Ningbo, China; ^2^Ministry of Agriculture Key Laboratory of Molecular Biology of Crop Pathogens and Insects, Institute of Insect Sciences, Zhejiang University, Hangzhou, China

**Keywords:** whitefly, endosymbionts, vertical transmission, host fitness, sex ratio

## Abstract

Insects commonly harbor maternally inherited intracellular symbionts in nature, and the microbial partners often exert influence on host reproduction and fitness to promote their prevalence. Here, we investigated composition of symbionts and their biological effects in the invasive *Bemisia tabaci* MED species of a whitefly complex. Our field surveys revealed that populations of the MED whitefly, in addition to the primary symbiont *Portiera*, mainly contain two secondary symbionts *Hamiltonella*, which is nearly fixed in the host populations, and *Cardinium* with infection frequencies ranging from 0 to 86%. We isolated and established *Cardinium*-positive and *Cardinium*-free whitefly lines with a similar nuclear genetic background from a field population, and compared performance of the two whitefly lines. The infection of *Cardinium* incurred significant fitness costs on the MED whitefly, including reduction of fecundity and egg viability as well as delay in development. We then selectively removed *Hamiltonella* from the *Cardinium*-free whitefly line and compared performance of two whitefly lines, one harboring both *Portiera* and *Hamiltonella* and the other harboring only *Portiera*. While depletion of *Hamiltonella* had little or only marginal effects on the fecundity, developmental rate, and offspring survival, the *Hamiltonella*-free whitefly line produced very few female offspring, often reducing the progeny female ratio from about 50% to less than 1%. Our findings indicate that the varying costs and benefits of the association between these two symbionts and the MED whitefly may play an important role in shaping their differential prevalence in the field.

## Introduction

Symbiotic microorganisms are associated with a broad range of invertebrates in nature. The symbionts are generally divided into primary and secondary symbionts based on that whether or not their association is essential for host development and survival (Douglas, 1989; Baumann, 2005). Insects regularly are associated with a variety of symbionts that confer specific benefits to hosts to keep the symbionts being maintained in host populations (Feldhaar, 2011; Douglas, 2015; Ayoubi et al., 2020). For example, in some pea aphids, “*Candidatus* Hamiltonella defensa” (hereafter *Hamiltonella*) provides protection against parasitoid wasps (Oliver et al., 2003; Oliver and Higashi, 2019). In addition, some symbionts including *Wolbachia* sp. and “*Candidatus* Cardinium hertigii” (hereafter *Cardinium*) manipulate host reproduction leading to variation of sex ratio in various haplodiploid insects and increasing fitness of infected females in diploid insects (Zchori-Fein et al., 2004; Werren et al., 2008; Nguyen et al., 2017). These symbionts are maintained through host generations by vertical transmission from parents to offspring and may vary in infection frequencies among host populations, possibly in association with manipulation of host reproduction and/or fitness (Turelli and Hoffmann, 1991; Harris et al., 2010; Himler et al., 2011; McLean et al., 2018).

*Bemisia tabaci* is a whitefly complex consisting of over 40 cryptic species, some of which are economically important pests such as the two widespread invasive species, tentatively named as Middle East Asia Minor 1 (MEAM1) and Mediterranean (MED) (Liu et al., 2007; De Barro et al., 2011; Hu et al., 2011; Shadmany et al., 2018; Misaka et al., 2020). These phloem feeding insects have a haplodiploid genetic system in which a fertilized egg develops into a diploid female and an unfertilized egg develops into a haploid male (Byrne and Bellows, 1991). The whiteflies harbor the primary symbiont “*Candidatus* Portiera aleyrodidarum” (hereafter *Portiera*) and seven known secondary symbionts, including *Hamiltonella* and *Cardinium* (Zchori-Fein and Brown, 2002; Weeks et al., 2003; Gottlieb et al., 2008; Bing et al., 2013a,b; Lei et al., 2021). In addition, these symbionts often contain some strains with genetic differentiations (Kanakala and Ghanim, 2019). *Portiera*, like some other primary symbionts of phloem-feeding insects, provide essential amino acids as well as carotenoids for its whitefly hosts (Sloan and Moran, 2012). The secondary symbionts have diverse effects on their hosts. For example, the *Rickettsia* confers general benefits to host fitness (Himler et al., 2011) and assists its host in resistance to entomopathogen *Pseudomonas syringae* in MEAM1 whitefly (Hendry et al., 2014); the *Hamiltonella* influences host sex ratio via provisioning of nutrients in MEAM1 whitefly (Shan et al., 2019; Wang Y. B. et al., 2020), and some *Cardnium* and *Rickettsia* strains confer lower host fitness in *B. tabaci* MED and SSA1-SG3 species, respectively (Fang et al., 2014; Ghosh et al., 2018; Zhao et al., 2018). In general, whiteflies of this species complex are infected with multiple symbionts and the dynamics of bacterial infections vary among host species/populations (Zchori-Fein et al., 2014). Yet, the factors causing these variations as well as the underlying mechanisms are poorly understood.

This study concerns the natural occurrence of whitefly secondary symbionts and the fitness costs/benefits associated with their infections in the *B. tabaci* whitefly, focusing on the invasive MED species, which is one group of notorious agricultural pests worldwide having developed high resistance to insecticide (Wang et al., 2020a,b). The MED whiteflies includes four phylogenetic clades Q1, Q2, Q3, and ASL, and Q1 is the most widely distributed genotype in most regions, especially in China (Chu et al., 2008; Gueguen et al., 2010; Gauthier et al., 2014). We surveyed symbionts in different geographical populations of MED in China, and found that the whitefly populations belong to the Q1 clade and were mainly infected with two secondary endosymbionts, *Cardinium* and *Hamiltonella*, with varying frequencies of infection among host populations. We then assessed the fitness costs/benefits associated with these two secondary symbionts by manipulating the presence/absence of the symbionts and comparing performance of whitefly populations with or without a given symbiont. Our results indicate that while *Cardinium* exerts some fitness cost on the host, *Hamiltonella* plays an essential role for the host to maintain a normal sex ratio, demonstrating diverse functions of different symbionts residing in the same host. We also discuss the association of the different functions of the two symbionts with their varying prevalence in natural host populations.

## Materials and Methods

### Whitefly Collection and Symbiont Identification

Field populations of *B. tabaci* were collected in different geographical locations in China from 2012 to 2014 (Supplementary Table 1). Total DNA was extracted from individual insect with Lysis buffer containing Tris–HCl, EDTA Non-idet P-40 and proteinase K as described previously (Shan et al., 2014). Each individual whitefly was ground in 50 μL of ice-cold lysis buffer by Grinding Mill and then incubated at 65°C for 2 h and 100°C for 10 min. Extractions were then centrifuged briefly and stored at 20°C. The species of *B. tabaci* were identified using *mitochondrial cytochrome oxidase I* (*mtCOI*) polymerase chain reaction restriction fragment-length polymorphism (PCR-RFLP) with the enzyme *Taq*I (Bosco et al., 2006), and further checked with *mtCOI* sequencing. All the *mtCOI* sequences generated in this work were deposited in NCBI GenBank.

The whiteflies identified as the MED species were used for detecting symbiont infection. The primary symbiont *Portiera* was first detected to confirm DNA quality. Then identifications of all seven known secondary symbionts including *Hamiltonella*, *Rickettsia, Cardinium*, *Wolbachia*, *Arsenophonus*, *Fritschea*, and *Hemipteriphilus* in the *B. tabaci* whiteflies were conducted using their specific PCR primers targeting the 16S rRNA or 23S rRNA gene and their sequences. The primer sequences of *mtCOI* and symbionts are listed in Supplementary Table 2. All the *16S rRNA* sequences generated in this work were deposited in NCBI GenBank.

### Isolation and Establishment of C^+^ and C^–^ Whitefly Lines

The MED population was originally collected from eggplants in Maoming, Guangdong Province, China in 2013 (population no. 11 in Supplementary Table 1), and maintained on cotton plants (*Gossypium hirsutum* cv. Zhe-Mian 1793) at 26°C, a photoperiod of 14:10 light/dark (L/D) and 60–80% relative humidity. The infection of *Cardinium* was 63.6% in the original population (Supplementary Table 1). The female lines of *Cardinium-*positive (C^+^) and *Cardinium*-free (C^–^) were isolated from the colony after testing by PCR. Briefly, 80 mated females were collected, and each female was reared in isolation on a cotton leaf, enclosed in a leaf-clip cage, to feed and oviposit for 7 days. The females were then collected and examined individually for the presence (C^+^) or absence (C^–^) of *Cardinium*. Twenty-five days later, the progenies of C^+^ females and those of C^–^ females were, respectively, pooled together and reared on new cotton plants in two separate insect rearing cages.

In consideration of the possible genetic heterogeneity between the two whitefly lines, we introgressed the C^–^ line into the C^+^ line over 6 generations to homogenize their nuclear background following the protocol as described by Himler et al. (2011). In every introgression, about 30 C^–^ males were mated with about 30 virgin C^+^ females. Subsequently C^–^ males were continuously mated to the introgressed virgin C^+^ female progenies for six consecutive generations. Thereafter, the introgressed C^+^ line and C^–^ line were used for comparing their fitness. We confirmed that the C^+^ line harbor the primary symbiont *Portiera*, and the two secondary symbionts *Hamiltonella* and *Cardinium*, and the C^–^ line harbor only *Portiera* and *Hamiltonella* prior to experiments.

### Observing Effects of *Cardinium* on Host Fitness and Vertical Transmission of the Symbiont

To examine the effects of *Cardinium* on host fitness and reproduction, we conducted four treatments of mating between the C^+^ and C^–^ lines including C^+^ ♀ × C^+^ ♂, C^–^ ♀ × C^–^ ♂, C^+^ ♀ × C^–^ ♂, and C^–^ ♀ × C^+^ ♂. In each replicate of a given mating treatment, a single virgin female and a single virgin male were reared together on a leaf of a cotton plant, enclosed in a leaf-clip cage, to feed, mate, and oviposit for 7 days. The adults were then removed and the eggs in each replicate were counted. After a further 10 days, the nymphs in each replicate were counted, and then on the 22nd, 27th, 32nd, and 37rd day since the removal of the females used in the mating treatments, adult progenies were collected, sexed, and counted. From these data, we were able to calculate the number of eggs laid per female, percentage of egg hatching, survival from the 1st instar nymph to adulthood, and number of adult progenies and their sex ratio. To observe the fidelity of vertical transmission of *Cardinium*, we randomly collected adult progenies from each of the four mating treatments to detect the presence of the bacterium using PCR with its specific primer (Supplementary Table 2).

### Antibiotic Treatment to Eliminate *Hamiltonella*

To assess effects of the other secondary symbiont *Hamiltonella* on the insect, we used a cocktail of antibiotics to specifically eliminate the bacterium from the C^–^ whitefly line which harbored only *Portiera* and *Hamiltonella*. This cocktail of antibiotics had been used successfully to remove this symbiont from pea aphids and from the MEAM1 species of the *B. tabaci* whitefly complex (Douglas et al., 2006; Tsuchida et al., 2010; Shan et al., 2019). Hundreds of adults (F0) were fed for 4 days with an artificial diet composed of ampicillin, gentamycin, and cefotaxime (each at 500 μg/ml) mixed with 25% sucrose, and the artificial diets were renewed every 2 days. The control sucrose diet solution was supplied without antibiotics. Immediately after the feeding, the whiteflies were transferred to cotton plants to feed and oviposit for 10 days, and then their progenies (F1) were maintained on the plants until they developed to adults in 30 days. To ameliorate the potentially direct effects of antibiotics on the insects, the offspring (F1) adults were used to test the presence of symbionts and conduct bioassays.

### Observing Effects of *Hamiltonella* on Host Reproduction and Vertical Transmission of the Symbiont

To observe effects of *Hamiltonella* on the reproduction of MED, we used F1 adults of the Control treatment (CK) where all individuals naturally harbored *Hamiltonella*, and those of F1 whiteflies of the antibiotic treatment (AT) that were depleted of *Hamiltonella* to conduct four treatments of mating: CK♀ × CK♂, AT♀ × AT♂, CK♀ × AT♂, and AT♀ × CK♂. In each replicate of a given mating treatment, a single virgin female and a single virgin male were reared together on a leaf of a cotton plant, enclosed in a leaf-clip cage, to feed, mate, and oviposit for 7 days. Then the female and male of the pair were collected and examined separately for the presence/absence of symbionts using qPCR. Adult progenies (F2) of the mating treatments were collected on the 22nd, 27th, 32nd, and 37rd day since the removal of F1 adults, sexed and counted. To observe the vertical transmission of *Hamiltonella*, we randomly collected some F2 progenies from each of the four mating treatments to detect the bacterium using qPCR, and detection of the *Portiera* of these whiteflies was also conducted as a reference.

### Assessing Effects of *Hamiltonella* on Host Fitness

Because F2 progenies produced by F1 whiteflies of the antibiotic treatment (AT) (that were depleted of *Hamiltonella*) were nearly all males, experiments to examine the effects of *Hamiltonella* on host fitness were not feasible with F2 whiteflies. We thus assessed the effects of *Hamiltonella* on host fitness using F1 adults in the following three treatments: (1) Unmated (CK), a single virgin female of the CK whitefly which was supposed to produce only male progenies; (2) CK, a female and a male of the CK whiteflies; (3) AT, a female and a male from the antibiotic treatment. The experimental protocol was the same as that used for observing the effects of *Cardinium* on host fitness, as described above.

### Determining Locations of Symbionts in Whitefly Hosts

The presence and localization of symbionts in the two whitefly lines were observed using fluorescence *in situ* hybridization analysis with the protocol of Shan et al. (2014). Insects were fixed in Carnoy’s fixative (ethanol/chloroform/glacial acetic acid, 6:3:1) overnight, decolorized in 6% H_2_O_2_ in ethanol for 2 h, 0.1% Triton X-100 for 1 h and hybridized overnight in hybridization buffer (20 mmol/lTris–HCl (pH 8.0), 0.9 mol/l NaCl, 0.01% sodium dodecyl sulfate, and 30% formamide) with 10 pmol of fluorescent probes/ml. The three symbionts *Portiera, Hamiltonella*, and *Cardinium* were detected with their specific probes BTP1-Cy3 (5′-Cy3-TGTCAG TGTCAGCCCAGAAG-3′) (Gottlieb et al., 2006), BTH-Cy5 (5′-Cy5-CCAGATTCCCAGACTTTACTCA-3′) (Gottlieb et al., 2008), and/or Card-Cy5 (5′-TATCAATTGCAGTTCTAGCG-3′) (Matalon et al., 2007), respectively.

### Quantitative PCR for Assessing Symbionts Densities

The quantity of symbionts was assessed using quantitative PCR (qPCR) with the SYBR^®^ Premix Ex Taq^TM^ (Takara) and Bio-Rad CFX96^TM^ Real-Time System. *Portiera* and *Hamiltonella* were determined with the *16S rRNA* gene, and the β*-actin* gene (nuclear gene) of whiteflies was measured in parallel for normalization. The relative density of the symbionts was defined as the ratio of bacterium single-copy genes to insect single-copy genes and calculated using the comparative CT method (2^–ΔΔCt^). The primer sequences are listed in Supplementary Table 2.

### Statistical Analysis

The data of developmental curves was analyzed via the Cox proportional-hazards model test, and other data of whitefly performance were analyzed using one-way analysis of variance (ANOVA), and Fisher’s least significant difference (LSD) tests were used for *ad hoc* multiple comparisons. All the data analyses were performed using SPSS 20.0 Statistics.

## Results

### Diversity of Symbionts in Field Whitefly Populations

**FIGURE 1:**
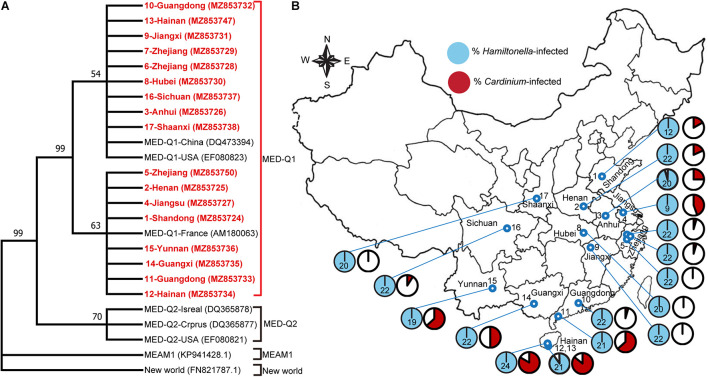
The infection of secondary symbionts in field MED whitefly populations sampled in various locations in China. **(A)** Phylogenetic tree of the different whitefly populations based on the *mtCOI* sequences (∼760 bp). Maximum Likelihood algorithms available in MEGA-X were used to infer phylogenetic relationships of the sequences that are shown as a cladogram. The *mtCOI* sequences of 17 MED populations from this study are indicated in bold red. The Genbank accession number is shown in bracket. Bootstrap values (>50%) are shown on branches. **(B)** Infection frequencies of *Cardinium* and *Hamiltonella* in various geographical populations. The numbers in the map indicated the whitefly population No. The number in each of the pie charts indicates the sample size for PCR screens in that population. For details of the sample collection information, see Supplementary Table 1.

We first analyzed genetic differentiation of the 17 field populations of MED whitefly based on *mtCOI* gene sequence. All of the MED populations belong to the subclade Q1 (Figure 1A). Subsequently, we surveyed the diversity of symbionts in these MED populations. In addition to the primary symbiont *Portiera*, three secondary symbionts were found. Among them, *Hamiltonella* showed high infection frequency, 100% in 15 populations and 95 and 90.5% in the two remaining populations, respectively (Figure 1B and Supplementary Table 1). Moreover, phylogenetic analysis of the 16S rRNA sequence show that all of the *Hamiltonella* belong to the similar strains (Supplementary Figure 1). *Cardinium* was detected in 13 of the 17 whitefly populations with infection frequencies varying from 4.5 to 85.7% (Figure 1B and Supplementary Table 1). *Cardinium* phylogenetic analyses indicated that the symbiont of *B. tabaci* were classified into four groups/strains (C1–C4) (Kanakala and Ghanim, 2019), and all of the tested *Cardinum* from the different populations clustered within the C4 group in the present study (Supplementary Figure 2). *Rickettsia* was detected in only one of the 17 whitefly population with an infection frequency of 12.5% (Supplementary Table 1).

### *Cardinium-*Positive (C^+^) and *Cardinium*-Free (C^–^) Whitefly Lines

**FIGURE 2:**
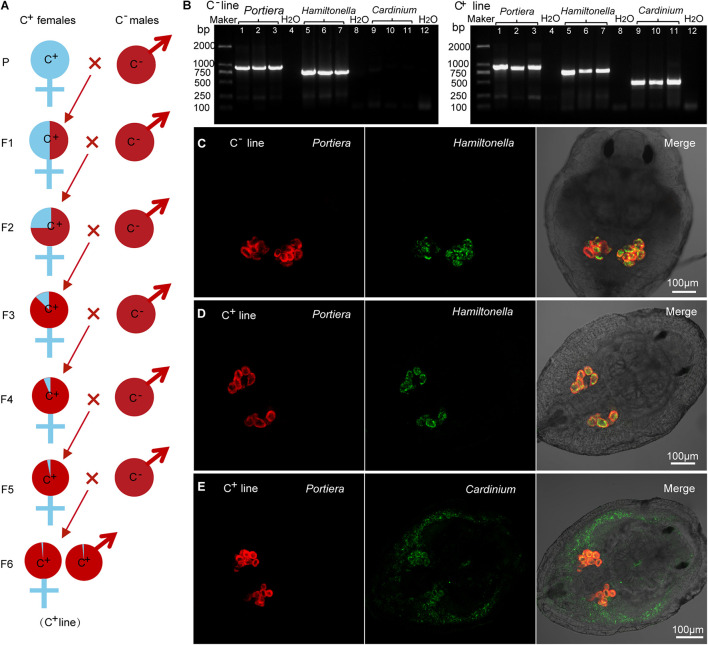
The presence/absence of symbionts in *Cardinium-*positive (C^+^) and *Cardinium-*free (C^–^) whitefly lines. **(A)** Establishment of C^+^ and C^–^ whitefly lines via introgression. The male C^–^ whiteflies were introduced to mate with female C^+^ whiteflies for 6 generations to homogenize their nuclear background. P: parental generation; F1–F6, the first to sixth filial generations. **(B)** *Portiera*, *Hamiltonella* and *Cardinium* were detected in C^+^ line, and *Portiera* and *Hamiltonella* but not *Cardinium* were detected in C^–^ line. Lane 1–3: *Portiera*; lane 5–7: *Hamiltonella*; lane 9–11: *Cardinium*; lane 4, 8, and 12: negative control (ddH_2_O). **(C)** The spatial distribution of *Portiera* (red) and *Hamiltonella* (green) in the C^–^ line whitefly. **(D)** The spatial distribution of *Portiera* (red) and *Hamiltonella* (green) in the C^+^ line whitefly. **(E)** The spatial distribution of *Portiera* (red) and *Cardinium* (green) in the C^+^ line whitefly.

We isolated *Cardinium-*positive (C^+^) and *Cardinium*-free (C^–^) individuals from field population no. 11 of MED (Figure 1B and Supplementary Table 1) and introgressed to establish C^+^ and C^–^ lines with a similar genetic background (Figure 2A). All individuals of the C^+^ line harbored *Portiera*, *Hamiltonella*, and *Cardinium*, while those of the C^–^ line harbored only *Portiera* and *Hamiltonella* (Figure 2B). *Portiera* and *Hamiltonella* were strictly located in the bacteriocytes in both lines of whiteflies (Figures 2C,D). While *Cardinium* were not restricted to any tissues or cells, with distribution in both the bacteriocytes and body cavity in the C^+^ line (Figure 2E).

### Effects of *Cardinium* on Whitefly Fitness

**FIGURE 3:**
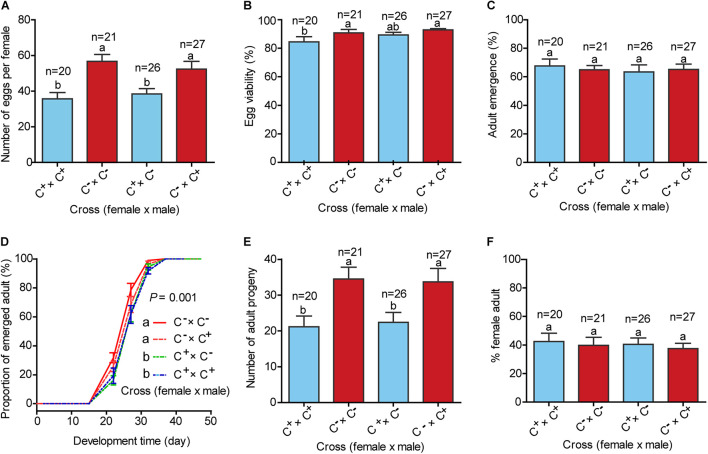
Life history parameters of four treatments of mating between C^+^ and C^–^ whiteflies. **(A)** Mean number of eggs per female produced in 7 days; **(B)** Percentage of eggs that hatched; **(C)** Proportion of survival from the 1st instar nymph to adulthood; **(D)** Development time of progenies from egg to adulthood, expressed as the cumulative proportion of adult emergence; **(E)** Number of progenies that survived to adulthood; **(F)** Sex ratio of the adults. C^+^: the MED whitefly line harboring *Portiera*, *Hamiltonelle* and *Cardinium*; C^–^: the MED whitefly line harboring *Portiera* and *Hamiltonelle* but not *Cardinium*. In each replicate of the four treatments of mating, a female and a male were reared together to mate and oviposit for 7 days. Developmental curves were analyzed with the Cox proportional-hazards model tests, and other data were analyzed with one-way ANOVA followed by LSD test for multiple comparisons. The data are mean ± SEM, and the different letters indicate significant differences at *P* < 0.05.

To investigate the effects of *Cardinium* on host fitness and reproduction, four treatments of mating were conducted between C^+^ and C^–^ lines including C^+^♀ × C^+^♂, C^–^♀ × C^–^♂, C^+^♀ × C^–^♂, and C^–^♀ × C^+^♂, and the fecundity and performance of progenies of the four mating treatments were assessed. The numbers of eggs produced by females in each of the two mating treatments C^+^♀ × C^+^♂ and C^+^♀ × C^–^♂ were significantly lower than females of C^–^♀ × C^–^♂ and C^–^♀ × C^+^♂ (Figure 3A; *F*_(__3_,_90__)_ = 7.225, *P* < 0.001). The hatchability of eggs of C^+^♀ × C^+^♂ was lower than those of the remaining three mating treatments, while the survival rates from the 1st instar nymph to adulthood did not differ significantly among the four mating treatments (Figures 3B,C; *F*_(__3_,_90__)_ = 2.445, *P* = 0.069 for egg viability; *F*_(__3_,_90__)_ = 0.155, *P* = 0.926 for adult emergence). The development times from egg to adult emergence of the progenies of the two mating treatments C^+^♀ × C^+^♂ and C^+^♀ × C^–^♂ were longer than those of C^–^♀ × C^+^♂ and C^–^♀ × C^–^♂ (Figure 3D; *P* = 0.001). The numbers of progenies that reached adulthood of the two mating treatments C^+^♀ × C^+^♂ and C^+^♀ × C^–^♂ were lower than those of C^–^♀ × C^+^♂ and C^–^♀ × C^+^♂ (Figure 3E; *F*_(__3_,_90__)_ = 4.645, *P* = 0.005), while the sex ratios of the four mating treatments were similar (Figure 3F; *F*_(__3_,_90__)_ = 0.173, *P* = 0.915).

Subsequently, we assessed the vertical transmission of *Cardinium* by investigating its infection in the progenies of the four treatments of mating between C^+^ and C^–^ lines. In the two mating treatments C^+^♀ × C^+^♂ and C^+^♀ × C^–^♂, where females were infected with *Cardinium*, nearly all individuals of their progenies were detected with *Cardinium*, while none of the progenies of C^–^♀ × C^+^♂ and C^–^♀ × C^–^♂, where females were not infected with *Cardinium*, were detected with this bacterium (Supplementary Figure 3). These observations indicate that *Cardinium* is transmitted from female parent to progeny with high fidelity, and the infection status of parent male plays little or no role in the vertical transmission.

### Effects of *Hamiltonella* on Whitefly Reproduction

**FIGURE 4:**
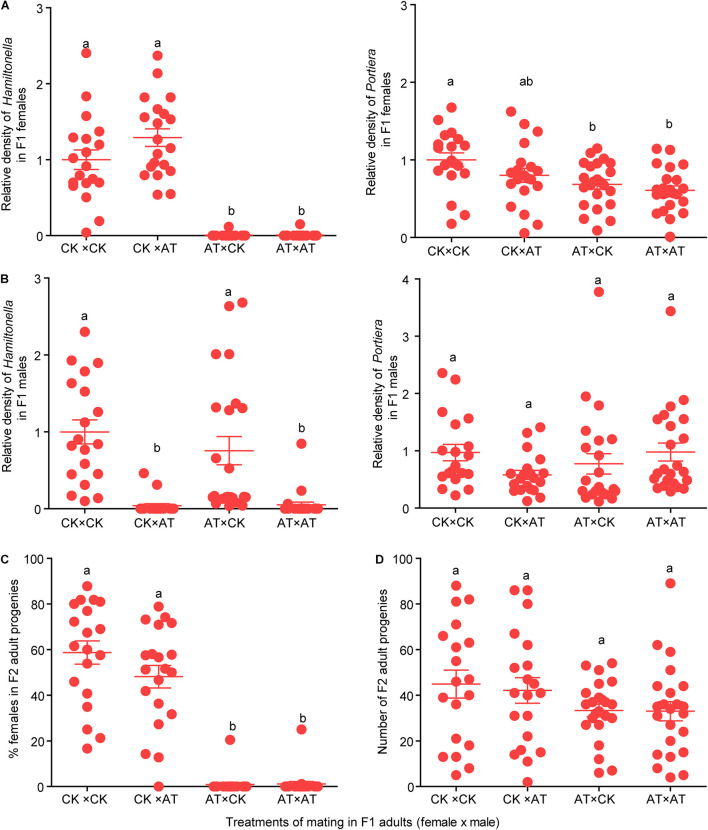
Effects of *Hamiltonella* on sex ratio and number of progenies. **(A,B)** Relative density of *Portiera* and *Hamiltonella* in F1 female **(A)** and male **(B)** adults used in the four crosses between CK (control) and AT (Antibiotics treatments). The relative density of the symbionts was measured as the ratio of bacterium single-copy *16S rRNA* genes to insect single-copy β*-actin* genes. **(C,D)** Percentage of female progenies **(C)** and number of adult progenies **(D)** of F2 produced by F1 adults in the four treatments of mating between CK and AT whiteflies. CK: Control; AT: F1 adult whiteflies produced by F0 adults that were fed with antibiotics for 4 days. The data are mean ± SEM, and the different letters indicate significant differences at *P* < 0.05 (One-way ANOVA followed by LSD test for multiple comparisons).

To investigate the effects of *Hamiltonella* on the reproduction of MED whitefly, four treatments of mating between antibiotic-treated and untreated F1 adult whiteflies were conducted, and the *Hamiltonella* status of F1 adults as well as the number and females % of F2 adult progenies were observed. In the F1 adults for mating experiments, the *Hamiltonella* were selectively depleted in both females and males using the antibiotic treatment (Figures 4A,B; *F*_(__3_,_81__)_ = 70.927, *P* < 0.001 for female; *F*_(__3_,_81__)_ = 15.296, *P* < 0.001 for male), while the abundance of *Portiera* was slightly reduced in females but not affected in males (Figures 4A,B; *F*_(__3_,_81__)_ = 5.136, *P* = 0.003 for female; *F*_(__3_,_81__)_ = 1.584, *P* = 0.200 for male). After the depletion of *Hamiltonella*, the female whiteflies produced extremely low percentage of female progenies, and the *Hamiltonella* status of the mating males had no significant effect on the sex ratio of the progenies (Figure 4C; *F*_(__3_,_81__)_ = 82.428, *P* < 0.001). However, two female whiteflies of F1 in the antibiotic treatment still harbored a low abundance of *Hamiltonella* (Figure 4A) and approximately 20% of their progenies were female (Figure 4C). In addition, the total number of progenies of *Hamiltonella*-free female whiteflies appeared slightly lower compared to that of untreated whiteflies (Figure 4D; *F*_(__3_,_81__)_ = 1.669, *P* = 0.180).

To determine the fidelity of vertical transmission of *Hamiltonella* in the host, effort was made to detect the bacterium in the progenies of the four treatments of mating between *Hamiltonella-*depleted whiteflies and non-antibiotic treated whiteflies. *Hamiltonella* was exclusively transmitted from the females to offspring, regardless of the *Hamiltonella* infection status of the mating males (Supplementary Figure 4).

### The Influence of *Hamiltonella* on Whitefly Fitness

**FIGURE 5:**
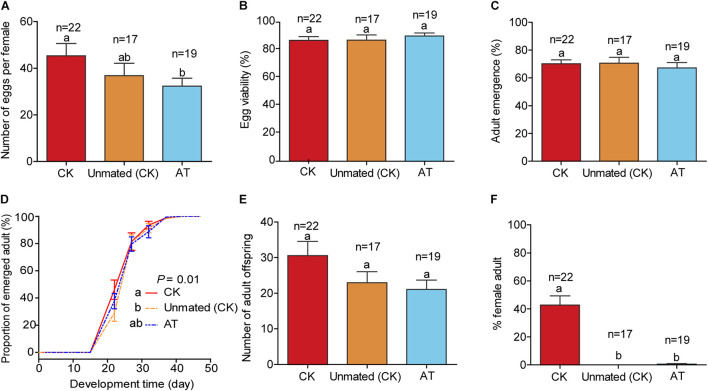
Life history parameters of *Hamiltonella*-positive and *Hamiltonella-*free whiteflies on cotton plants. **(A)** Mean number of eggs per F1 female produced in 7 days; **(B)** Percentages of eggs that hatched; **(C)** Proportion of survival from the 1st instar nymph to adulthood; **(D)** Development time of progeny from egg to adulthood, expressed as the cumulative proportion of adult emergence; **(E)** The number of progenies that survived to adulthood; **(F)** Sex ratio of the adults. Unmated (CK): untreated virgin female adults; CK: untreated but mated females; AT: antibiotics-treated and mated females. In each replicate of Unmated CK, a female was allowed to oviposit for 7 days, and in each replicate of CK and AT, a female and a male were reared together to mate and oviposit for 7 days. The data are mean ± SEM, and the different letters indicate significant differences at *P* < 0.05.

To assess other fitness costs/benefits of *Hamiltonella* on the whitefly host, we compared the performance of CK whiteflies (untreated and mated), unmated CK females (untreated and unmated), and AT whiteflies (*Hamiltonella*-depleted by antibiotic treatment). The three treatments did not differ significantly in egg viability, % adult emergence, and number of progenies (Figures 5B,C,E; *F*_(__2_,_55__)_ = 0.406, *P* = 0.668 for egg viability; *F*_(__2_,_55__)_ = 0.253, *P* = 0.777 for adult emergence; *F*_(__2_,_55__)_ = 2.258, *P* = 0.114 for progeny size). The number of eggs produced per *Hamiltonella*-depleted female was significantly lower than that of CK females, but did not differ significantly from that of unmated CK females (Figure 5A; *F*_(__2_,_55__)_ = 2.306, *P* = 0.109). Similarly, the development rate of progenies produced by *Hamiltonella*-depleted females was slightly lower than that of mated CK females, but did not differ significantly from that of unmated CK females (Figure 5D; *P* = 0.266). However, while 42% of the progenies produced by CK females were female, only 0.6% of the progenies produced by the *Hamiltonella*-depleted females were female, and none of the progenies produced by the unmated CK females was female as might be expected (Figure 5F; *F*_(__2_,_55__)_ = 33.352, *P* < 0.001).

## Discussion

The maternally inherited symbionts induced hosts to increase production of infected daughters by enhancing fitness or manipulating reproduction to facilitate the spread of symbiotic microorganisms in host populations. In this study, we surveyed the infection of two secondary symbionts *Cardinium* and *Hamiltonella* in field populations of the MED whitefly, and examined the effects of these two symbionts on the performance and reproduction of the whitefly host. Because the infection of *Hamiltonella* was essential to the host to survive normally through multiple generations, it was not feasible to create a MED whitefly line free of *Hamiltonella* but infected with *Cardinium* for the experiments. Nevertheless, the two MED whitefly lines used in the experiments for examining the functions of *Cardinium* differed only in the presence/absence of this symbiont, and the data obtained may be explored to understand the functions of the symbiont.

Our data show that *Cardinium* occurred widely in many field populations of the MED whitefly, but the infection frequencies were comparatively low and varied widely (Figure 1B and Supplementary Table 1). *Cardinium* is a widespread reproductive parasite and has been reported to induce parthenogenesis, cytoplasmic incompatibility and feminization in various arthropods (Weeks et al., 2001; Zchori-Fein et al., 2001, 2004; Hunter et al., 2003). These reproductive manipulations skew reproduction to favor infected females specifically and then boost the spread of the symbiont in their insect hosts (Turelli and Hoffmann, 1991; Engelstädter and Telschow, 2009; Harris et al., 2010; Hurst and Frost, 2015). Our laboratory experiments show that, while *Cardinium* is strictly vertically transmitted in the MED whitefly (Supplementary Figure 3), its infection as well as mating with uninfected lines do not affect the host sex ratio of this haplodiploid insect (Figure 3F), indicating that this bacterium in the MED whiteflies does not act as a reproductive parasite. Therefore, reproductive manipulation is unlikely a major factor affecting the spread of *Cardinium* in field populations of its whitefly host, which is consistent with previous reports (Fang et al., 2014).

However, the infection of *Cardinium* incurred significant fitness costs on the MED whitefly, including reduction of fecundity and egg viability as well as delay in development of immature stages (Figure 3). Fecundity, egg viability, and development are biological features that often vary with host and symbiont genotypes as well as environmental conditions. There could be other fitness benefits/costs associated with the infection of *Cardinium* that were not examined in this study. For example, a recent study provides some preliminary indication indicates that *Cardinium* may increase the thermal tolerance of MED whitefly, which is likely associated with host genetic background (Yang et al., 2021). Considering the high temperature in southern China, thermal tolerance could be an important factor responsible for the high prevalence of *Cardinium* in some southern provinces, i.e., Yunnan, Guangxi, Guangdong, and Hainan (Figure 1B). In addition, in another symbiosis between parasitoid *Encarsia inaron* and *Cardinium*, while the *Cardinium* reduces initial fecundity, it also increases the host *E. inaron* longevity which may mitigate some of this fecundity cost (White et al., 2011). This, together with the negative and/or benefit association of these biological features under various conditions with the infection of *Cardinium*, seem to explain the comparatively low but widely varying infection frequencies of this symbiont in the field (Figure 1B and Supplementary Table 1).

The genus of *Hamiltonella* is mainly found in whiteflies and their related groups, including aphids and psyllids (Haynes et al., 2003; Russell et al., 2003). *Hamiltonella* confers resistance to parasitic wasps in some aphids (Oliver et al., 2003; Moran et al., 2005). However, in whiteflies, the *Hamiltonella* lost the virulent genes associated with this defensive function but contains genes that are involved in the production of essential nutrients in its genome (Rao et al., 2012; Rollat-Farnier et al., 2015). In whiteflies of the *B. tabaci* complex, *Hamiltonella* is mostly associated with the two widespread invasive species MEAM1 and MED in both their native regions of the Middle East and Mediterranean and regions of their invasion around the globe (Chiel et al., 2007; Gueguen et al., 2010; Bing et al., 2013a; Parrella et al., 2014; Zchori-Fein et al., 2014), and occasionally is found in some populations of the indigenous species New World 1 and New World 2 (De Marchi et al., 2018; Wang et al., 2019).

In the present study, the selective elimination of *Hamiltonella* from the MED whitefly resulted in strongly biased sex ratios, with an excess of male progenies (Figures 4A–C). This abnormal sex ratio has also been observed in the MEAM1 whitefly after *Hamiltonella* was experimentally depleted and was found to be associated with failure of egg fertilization rather than with failure of copulation (Shan et al., 2019), and in the geenhouse whitefly *Trialeurodes vaporariorum* after the removal of the symbiont *Arsenophonus* (Wang Y. B. et al., 2020). Both *Hamiltonella* and *Arsenophonus* contain genes that are capable of synthesizing B vitamins (Rollat-Farnier et al., 2015; Santos-Garcia et al., 2018). The removal of these symbionts reduced B vitamin levels and inhibited fertilization for the two whiteflies, and dietary B vitamin supplementation rescued fitness of the hosts (Wang Y. B. et al., 2020). In addition, the symbionts *Wigglesworthia* and *Wolbachia*-produced B vitamins are significant for sexual maturation and reproduction in their diploid hosts tsetse flies and planthoppers, respectively (Snyder and Rio, 2015; Ju et al., 2020). In whiteflies, the B vitamin deficiency may influence the quality of eggs and sperm to prevent fertilization which results in a male-biased sex ratio in the haplodiploid insects. Thus, these symbionts are likely to provide the nutrients to benefit their whitefly host by influencing host sex allocation (Bondy and Hunter, 2019).

Relevant to these considerations is the impact of the male-dominated sex ratio of *Hamiltonella*-free whiteflies on the prevalence of *Hamiltonella* in field whitefly populations. Where investigated, natural populations of *B. tabaci* MEAM1 and the subclade Q1 of *B. tabaci* MED have a high prevalence of this bacterium (Gueguen et al., 2010; Zchori-Fein et al., 2014), suggesting that *Hamiltonella* is transmitted vertically with high fidelity. However, failure of transmission occurs occasionally under adverse field conditions, especially high temperatures, because *Hamiltonella* has a greater sensitivity to high temperatures than its insect host (Wernegreen, 2012; Shan et al., 2014, 2017). Recovery of a symbiont is generally argued to be mediated by horizontal transmission, a process that occurs with exceptionally low frequency for *Hamiltonella* (Darby and Douglas, 2003; Oliver et al., 2014; Smith et al., 2015). However, a related possibility is that the production of male-dominated offspring by a *Hamiltonella*-free female whitefly would facilitate re-acquisition of *Hamiltonella* in natural populations with an intermediate prevalence of *Hamiltonella*. The greater the bias toward male offspring in a *Hamiltonella*-free female, the greater the probability that these males would mate with *Hamiltonella*-positive females yielding *Hamiltonella*-positive offspring. An alternative set of explanations for the prevalence of *Hamiltonella* is that the symbiont became incorporated into the host reproductive process over time, so that the loss of the symbiont is now detrimental to the host and may eventually cause the collapse of the host population due to overproduction of males. This outcome is in the selective interest of *Hamiltonella*, and also the insect if the host benefits from this interaction.

It is widely recognized that host genotype can greatly affect the benefits/costs conferred by a symbiont infection (Cass et al., 2016). Populations of *B. tabaci* MED consist of four phylogenetic clades. In theory, even small genetic variations within a particular clade of the host might still affect the benefits/costs associated with a symbiont infection. In addition, the symbiont *Cardinium* has been reported to consist of four subclades in *B. tabaci* (Kanakala and Ghanim, 2019). Thus, while the results of this study have some general implications in the ecology of the MED whitefly in China because Q1 is the predominant, widely distributed phylogenetic clade in this country, caution needs to be exerted when these implications are extended to understand the symbiotic association of the MED whitefly with *Cardinium* and *Hamiltonella* in other host populations and geographic regions.

Taken together, The data show that field populations of the *B. tabaci* MED whitefly in China are often coinfected with two secondary symbionts *Cardinium* and *Hamiltonella*, which differ in biological roles in the host. The beneficial and cost interactions of the co-infections with host are likely to play a critical role in determining the distribution of the symbionts in natural populations. Further research on the biological effects across different microbial symbionts would contribute to our understanding of the selective factors influencing the long-term maintenance and coevolution of multiple maternally inherited symbionts in whiteflies and other insects.

## Data Availability Statement

The original contributions presented in the study are included in the article/Supplementary Material, further inquiries can be directed to the corresponding author.

## Author Contributions

Both authors conceived and designed the study, wrote the manuscript, contributed to the article, and approved the submitted version. H-WS conducted the experimental work and data analysis.

## Conflict of Interest

The authors declare that the research was conducted in the absence of any commercial or financial relationships that could be construed as a potential conflict of interest.

## Publisher’s Note

All claims expressed in this article are solely those of the authors and do not necessarily represent those of their affiliated organizations, or those of the publisher, the editors and the reviewers. Any product that may be evaluated in this article, or claim that may be made by its manufacturer, is not guaranteed or endorsed by the publisher.

## Supplementary Material

Supplementary Figure 1 Phylogenetic position of *Hamiltonella* of different insect hosts based on the *16S rRNA* sequences (∼720 bp). Maximum likelihood algorithms available in MEGA-X were used to infer phylogenetic relationships of the sequences that are shown as a cladogram. *Hamiltonella* sequences of 16 MED populations from this study are indicated in red. The Genbank accession number is shown in bracket. Bootstrap values (>50%) are shown on branches.

Supplementary Figure 2 Phylogenetic position of *Cardinium* of different whitefly populations based on the *16S rRNA* sequences (∼390 bp). Maximum likelihood algorithms available in MEGA-X were used to infer phylogenetic relationships of the sequences that are shown as a cladogram. *Cardinium* sequences of 12 MED populations from this study are indicated in red. The Genbank accession number is shown in bracket. Bootstrap values (>50%) are shown on branches.

Supplementary Figure 3 Vertical transmission of *Cardinium*. **(A)** PCR detection of *Cardinium* in the progenies of four treatments of mating. **(B)** The transmission rates of *Cardinium* in the progenies of the four treatments of mating. The numbers above the columns indicate the positive total number of tested samples.

Supplementary Figure 4 Vertical transmission of *Hamiltonella*. Relative density of *Hamiltonella* **(A)** and *Portiera* **(B)** in F2 progeny produced by F1 adults in the four treatments of mating between CK (control) and AT (Antibiotics treatments). The data are mean ± SEM, and the different letters indicate significant differences at *P* < 0.05 (One-way ANOVA followed by LSD test for multiple comparisons).
