# Supplementary material for: The Costs and Benefits of Two Secondary Symbionts in a Whitefly Host Shape Their Differential Prevalence in the Field
Source: Front Microbiol. 2021 Sep 30;12:739521. doi: 10.3389/fmicb.2021.739521 (PMC8515054; doi:10.3389/fmicb.2021.739521)
Supplement: Supplementary file 1 [file Data_Sheet_1.docx]

| Population  No. | Total no. tested | *Hamilto-nella* (%) | *Rickettsia* (%) | *Cardinium* (%) | Date of Collection | Host plants | Locality | (Latitude & Longitude) |
| --- | --- | --- | --- | --- | --- | --- | --- | --- |
| 1 | 12 | 100 | 0.0 | 16.7 | Jul 2012 | Eggplant | Jinan, Shandong | 36°33'N, 117°11'E |
| 2 | 22 | 100.0 | 0.0 | 18.2 | Aug 2012 | Eggplant | Zhoukou, Henan | 33°15'N, 114°27'E |
| 3 | 20 | 95.0 | 0.0 | 25.0 | Jul 2012 | Eggplant | Hefei, Anhui | 31°57'N,  117°28'E |
| 4 | 9 | 100.0 | 0.0 | 44.4 | Aug 2012 | Sweet potato | Nanjing, Jiangsu | 32°01’N,  118°32’E |
| 5 | 22 | 100.0 | 0.0 | 4.5 | Sept 2012 | Eggplant | Hangzhou, Zhejiang | 30°12'N,  120°06'E |
| 6 | 22 | 100.0 | 0.0 | 4.5 | Oct 2014 | Turnip | Hangzhou, Zhejiang | 30°12'N,  120°10'E |
| 7 | 22 | 100.0 | 0.0 | 0.0 | Jul 2012 | Gerbera | Haining, Zhejiang | 30°14'N,  120°11'E |
| 8 | 20 | 100.0 | 0.0 | 0.0 | Sept 2012 | Sweet potato | Wuhan, Hubei | 30°25'N,  114°16'E |
| 9 | 22 | 100.0 | 0.0 | 0.0 | Aug 2012 | Eggplant | Jiujiang, Jiangxi | 29°45'N,  115°47'E |
| 10 | 22 | 100.0 | 0.0 | 4.5 | Sept 2014 | Sweet potato | Guangzhou, Guangdong | 23°13'N, 113°15'E |
| 11 | 22 | 100.0 | 0.0 | 63.6 | Jun 2013 | Eggplant | Maoming, Guangdong | 22°16'N, 112°26'E' |
| 12 | 21 | 90.5 | 0.0 | 85.7 | Aug 2012 | Cucumber | Sanya, Hainan | 18°14'N  109°25'E |
| 13 | 24 | 100.0 | 12.5 | 83.3 | Aug 2014 | Eggplant | Sanya, Hainan | 18°11'N  109°15'E |
| 14 | 22 | 100.0 | 0.0 | 50.0 | Aug 2012 | Loofah | Nanning, Guangxi | 22°22'N,  108°13'E |
| 15 | 19 | 100.0 | 0.0 | 63.2 | Jul 2012 | Poinsettia | Kunming, Yunnan | 25°03'N,  102°42' |
| 16 | 22 | 100.0 | 0.0 | 9.1 | Jul 2012 | Eggplant | Chengdu, Sichuan | 30°42'N,  104°03'E |
| 17 | 20 | 100.0 | 0.0 | 0.0 | Jul 2012 | Sweet potato | Yangling, Shanxi | 34°11'N, 107°34'E |

**Table S1** Infection frequencies of secondary symbionts in various geographical populations of *B. tabaci* MED sampled in China.

Note: Each of the 17 whitefly populations was examined for the presence/absence of the eight symbionts reported so far in whiteflies of the *B. tabaci* complex. In this table, only the data on the secondary symbionts that were detected in these populations are presented, and the data on those symbionts that were undetected are omitted.

| Organism | Target gene | Primer name | Primer sequences (5’→3’) | Length (bp) | Tm (°C) | References |
| --- | --- | --- | --- | --- | --- | --- |
| **PCR** |  |  |  |  |  |  |
| *Portiera* | 16S rRNA | Por-F  Por-R | GGAAACGTACGCTAATAC  TGACGACAGCCATGCAGCAC | ~900 | 58 | Thierry et al., 2011 |
| *Hamiltonella* | 16S rRNA | Ham-F  Ham-R | TGAGTAAAGTCTGGGAATCTGG  AGTTCAAGACCGCAACCTC | ~750 | 58 | Zchori-Fein & Brown, 2002 |
| *Rickettsia* | 16S rRNA | Ric-F  Ric-R | GCTCAGAACGAACGCTATC  GAAGGAAAGCATCTCTGC | ~900 | 58 | Gottlieb et al., 2006 |
| *Cardinium* | 16S rRNA | CFB-F  CFB-R | GCGGTGTAAAATGAGCGTG  ACCTMTTCTTAACTCAAGCCT | ~450 | 58 | Weeks et al., 2003) |
| *Wolbachia* | 16S rRNA | Wol-F  Wol-R | TTGTAGCCTGCTATGGTATAACT  GAATAGGTATGATTTTCATGT | ~900 | 55 | O'Neill et al., 1992 |
| *Arsenophonus* | 23S rRNA | Ars23S-1  Ars23S-2 | CGTTTGATGAATTCATAGTCAAA  GGTCCTCCAGTTAGTGTTACCCAAC | ~600 | 58 | Thao et al., 2004 |
| *Fritschea* | 23S rRNA | U23F  23SIGR | GATGCCTTGGCATTGATAGGCGATGAAGGA  TGGCTCATCATGCAAAAGGCA | ~600 | 58 | Everett et al., 2005 |
| *Hemipteriphilus* | 16S rRNA | OLO-F  OLO-R | GCTCAGAACGAACGCTRKC  TTCGCCACTGGTGTTCCTC | ~670 | 58 | Bing et al., 2013 |
| *B. tabaci* | *mtCOI* | COI-F  COI-R | TGRTTYTTTGGTCATCCVGAAGT  TTACTGCACTTTCTGCCACATTAG | ~850 | 55 | This study |
| **qPCR** |  |  |  |  |  |  |
| *Portiera* | 16S rRNA | Port73-F  Port266-R | GTGGGGAATAACGTACGG  CTCAGTCCCAGTGTGGCTG | ~200 | 60 | Caspi-Fluger et al., 2011 |
| *Hamiltonella* | 16S rRNA | H-16S-Fis  H-16S-Ris | GCATCGAGTGAGCACAGTTT  TATCCTCTCAGACCCGCTAGA | ~240 | 60 | Brumin et al., 2011 |
| *Rickettsia* | *gltA* | glt375-F  glt574-R | TGGTATTGCATCGCTTTGGG  TTTCTTTAAGCACTGCAGCACG | ~200 | 60 | Caspi-Fluger et al., 2011 |
| *B. tabaci* | *β-actin* | Actin-F  Actin-R | TCTTCCAGCCATCCTTCTTG  CGGTGATTTCCTTCTGCATT | ~200 | 60 | Sinisterra et al., 2005 |

**Table S2** Primers used for PCR and qPCR

**References**

Bing, X. L., Yang, J., Zchori-Fein, E., Wang, X. W., Liu, S. S. (2013). Characterization of a newly discovered symbiont of the whitefly *Bemisia tabaci* (Hemiptera: Aleyrodidae). *Appl. Environ. Microbiol.* 79, 569-575. doi: 10.1128/AEM.03030-12

Brumin, M,, Kontsedalov, S., Ghanim, M. (2011). *Rickettsia* influences thermotolerance in the whitefly *Bemisia tabaci* B biotype. *Insect Sci.* 18, 57-66. doi:10.1111/j.1744-7917.2010.01396.x

Caspi-Fluger, A., Inbar, M., Mozes-Daube, N., Mouton, L., Hunter, M. S., Zchori-Fein, E. (2011). *Rickettsia* ‘in’and ‘out’: two different localization patterns of a bacterial symbiont in the same insect species. *PLoS One* 6, e21096. doi:10.1371/journal.pone.0021096

Everett, K. D., Thao, M., Horn. M., Dyszynski. G. E., Baumann, P. (2005). Novel chlamydiae in whiteflies and scale insects: endosymbionts '*Candidatus* Fritschea bemisiae' strain Falk and '*Candidatus* Fritschea eriococci' strain Elm*. Int. J. Syst. Evol. Microbiol*. 55, 1581-1587. doi:10.1099/ijs.0.63454-0

Gottlieb, Y., Ghanim, M., Chiel, E., Gerling, D., Portnoy, V., Steinberg, S. *et al*. (2006). Identification and localization of a *Rickettsia* sp. in *Bemisia tabaci* (Homoptera: Aleyrodidae). *Appl. Environ. Microbiol.* 72, 3646-3652. doi: 10.1128/AEM.72.5.3646-3652.2006

O'Neill, S.L., Giordano, R., Colbert, A. M. E., Karr, T. L., Robertson, H. M. (1992). 16S rRNA phylogenetic analysis of the bacterial endosymbionts associated with cytoplasmic incompatibility in insects. *Proc. Natl. Acad. Sci. USA* 89, 2699-2702. doi: 10.1073/pnas.89.7.2699

Sinisterra, X. H., McKenzie, C. L., Hunter, W. B., Powell, C. A., Shatters, R. G. Jr. (2005). Differential transcriptional activity of plant-pathogenic begomoviruses in their whitefly vector (*Bemisia tabaci*, Gennadius: Hemiptera Aleyrodidae). *J. Gen. Virol.* 86, 1525-1532. doi:10.1099/vir.0.80665-0

Thao M.L., Baumann, P. (2004). Evidence for multiple acquisition of *Arsenophonus* by whitefly species (Sternorrhyncha: Aleyrodidae). *Curr. Microbiol.* 48, 140-144. doi:10.1099/vir.0.80665-0

Thierry, M., Becker, N., Hajri, A., Reynaud, B., Lett, J. M., Delatte, H. (2011). Symbiont diversity and non-random hybridization among indigenous (Ms) and invasive (B) biotypes of *Bemisia tabaci*. *Mol. Ecol.* 20, 2172-2187. doi: 10.1111/j.1365-294X.2011.05087.x

Weeks, A.R., Velten, R., Stouthamer, R. (2003). Incidence of a new sex-ratio-distorting endosymbiotic bacterium among arthropods. *Proc. R. Soc. Lond. B* 270, 1857-1865. doi: 10.1098/rspb.2003.2425

Zchori-Fein, E., Brown, J. (2002). Diversity of prokaryotes associated with *Bemisia tabaci* (Gennadius)(Hemiptera: Aleyrodidae). *Ann. Entomol. Soc. Am.* 95, 711-718. doi: 10.1603/0013-8746(2002)095[0711:DOPAWB]2.0.CO;2
