# Supplementary figures and images for: The Costs and Benefits of Two Secondary Symbionts in a Whitefly Host Shape Their Differential Prevalence in the Field
Source: Front Microbiol. 2021 Sep 30;12:739521. doi: 10.3389/fmicb.2021.739521 (PMC8515054; doi:10.3389/fmicb.2021.739521)

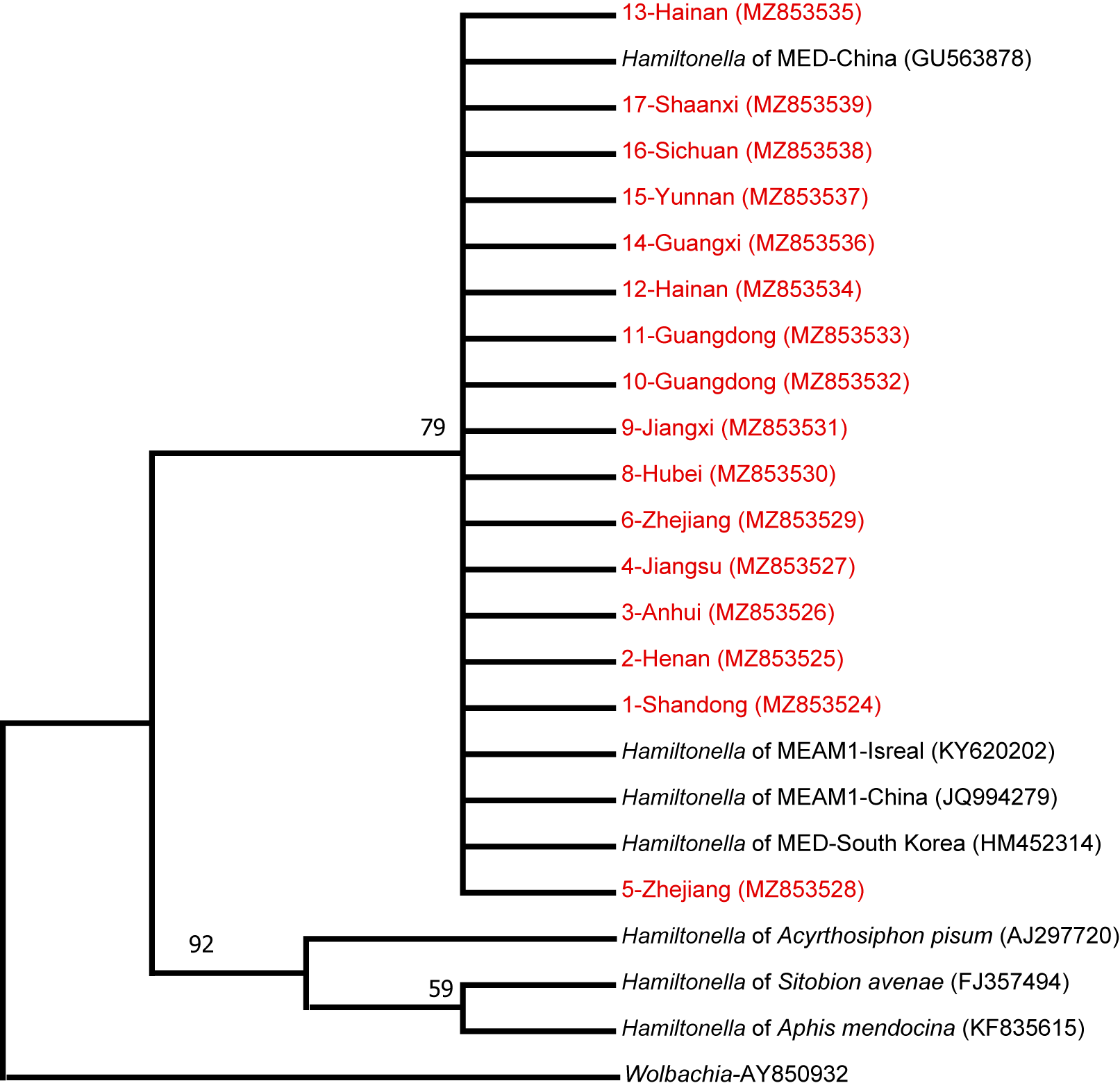

Supplement: Supplementary Figure 1 — Phylogenetic position of Hamiltonella of different insect hosts based on the 16S rRNA sequences (∼720 bp). Maximum likelihood algorithms available in MEGA-X were used to infer phylogenetic relationships of the sequences that are shown as a cladogram. Hamiltonella sequences of 16 MED populations from this study are indicated in red. The Genbank accession number is shown in bracket. Bootstrap values (>50%) are shown on branches. [file Image_1.TIF]

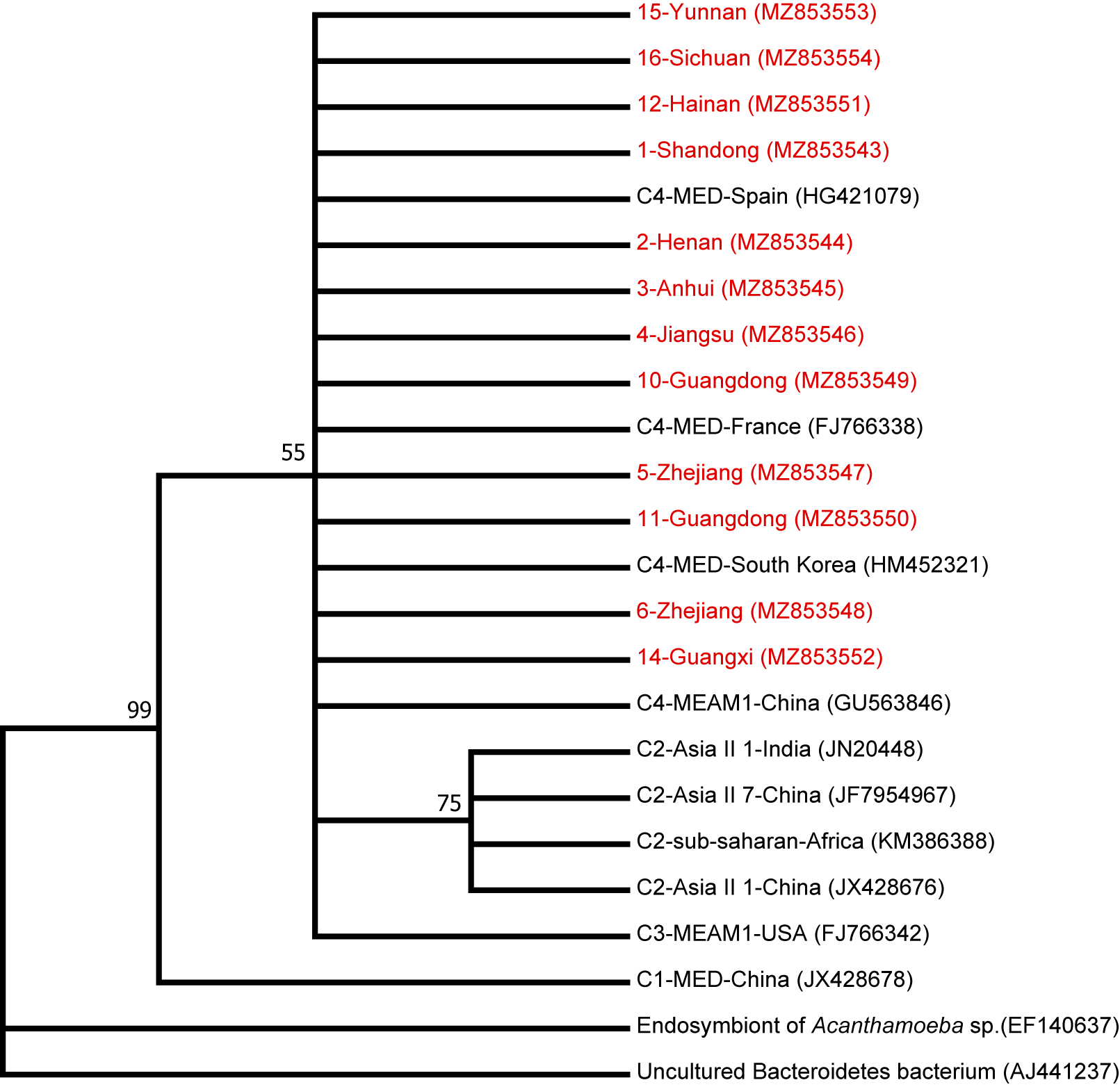

Supplement: Supplementary Figure 2 — Phylogenetic position of Cardinium of different whitefly populations based on the 16S rRNA sequences (∼390 bp). Maximum likelihood algorithms available in MEGA-X were used to infer phylogenetic relationships of the sequences that are shown as a cladogram. Cardinium sequences of 12 MED populations from this study are indicated in red. The Genbank accession number is shown in bracket. Bootstrap values (>50%) are shown on branches. [file Image_2.TIF]

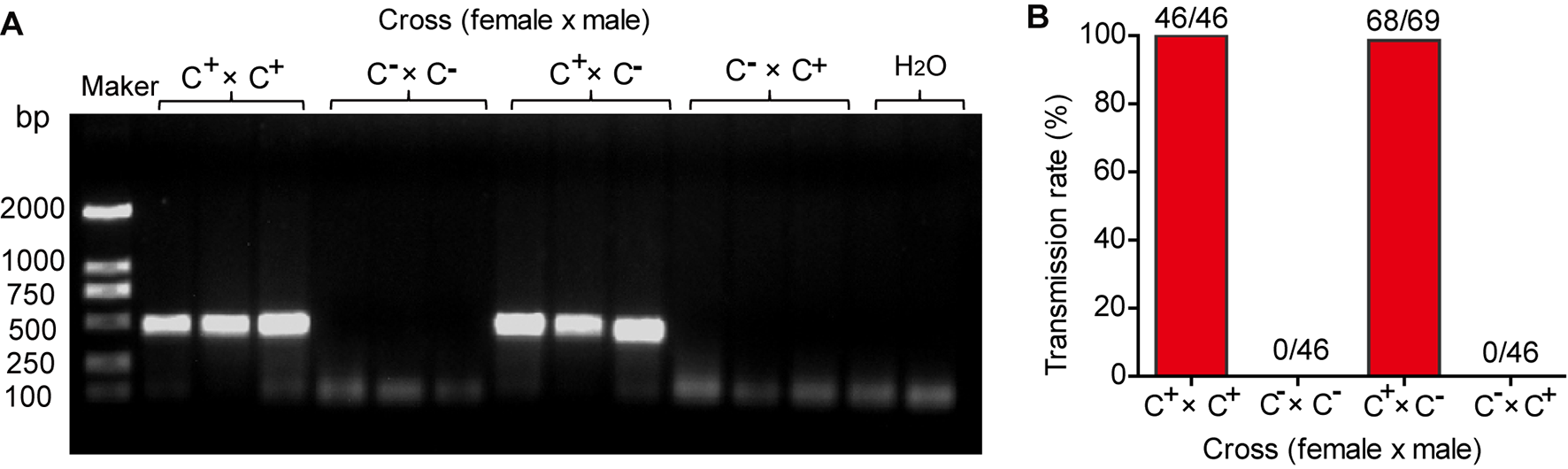

Supplement: Supplementary Figure 3 — Vertical transmission of Cardinium. (A) PCR detection of Cardinium in the progenies of four treatments of mating. (B) The transmission rates of Cardinium in the progenies of the four treatments of mating. The numbers above the columns indicate the positive total number of tested samples. [file Image_3.TIF]

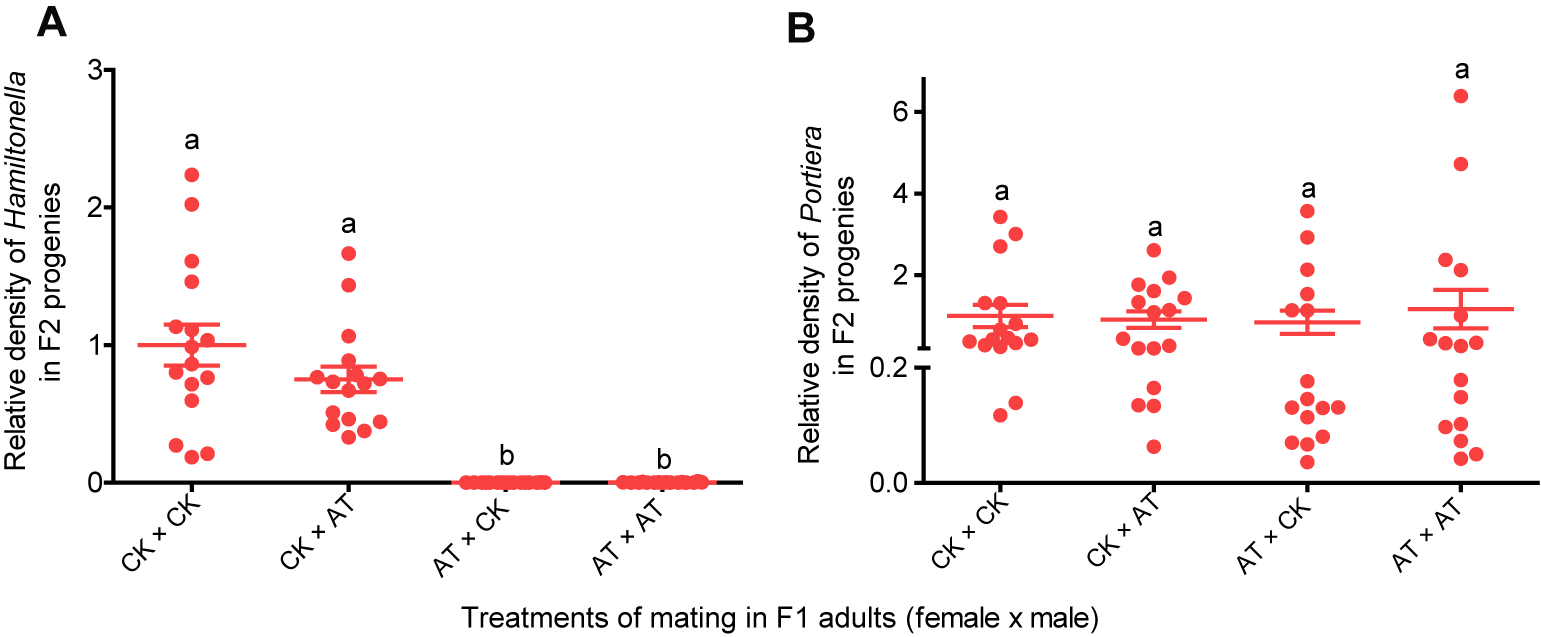

Supplement: Supplementary Figure 4 — Vertical transmission of Hamiltonella. Relative density of Hamiltonella (A) and Portiera (B) in F2 progeny produced by F1 adults in the four treatments of mating between CK (control) and AT (Antibiotics treatments). The data are mean ± SEM, and the different letters indicate significant differences at P < 0.05 (One-way ANOVA followed by LSD test for multiple comparisons). [file Image_4.TIF]
